# Heat variation on MHD Williamson hybrid nanofluid flow with convective boundary condition and Ohmic heating in a porous material

**DOI:** 10.1038/s41598-023-33043-z

**Published:** 2023-04-13

**Authors:** Ahmed M. Rashad, Mohamed A. Nafe, Dalia A. Eisa

**Affiliations:** 1 Department of Mathematics, Faculty of Science, Aswan University, Aswan, 81528 Egypt; 2 Department of Mathematics, Faculty of Science, New Valley University, Al-Kharga, 72511 Al-Wadi Al-Gadid Egypt

**Keywords:** Engineering, Mathematics and computing, Nanoscience and technology, Physics

## Abstract

The aim of the present study is to explore the variation of heat on MHD Williamson hybrid nanofluid (Ag-TiO_2_/H_2_O) model for steady two-dimensional and incompressible flow with a convective boundary condition in a curved coordinate porous system with Ohmic heating. Nusselt number is distinguished by the process of thermal radiation. The partial differential equations are controlled by the curved coordinate’s porous system, which depicts the flow paradigm. Employing similarity transformations, the acquired equations were turned into coupled non-linear ordinary differential equations. The governing equations were disbanded by RKF45 via shooting methodology. The focus is on examining physical characteristics such as heat flux at the wall, temperature distribution, velocity of flow, and surface friction coefficient for a variety of related factors. The analysis explained that increasing permeability, Biot and Eckert numbers enhance temperature profile and slowdown heat transfer. Moreover, convective boundary condition and thermal radiation enhance the friction of the surface. The model is prepared as an implementation for solar energy in processes of thermal engineering. Morever, this research has enormous applications in the industries of polymer and glass, also in the field of heat exchangers styling, cooling operations of metallic plates, etc.

## Introduction

Due to constraints in the applications of Newtonian fluids, the study of non-Newtonian fluids has grown in relevance in modern research. Non-Newtonian fluids include honey, starch, sprays of lubricating, ketchup and hydraulic liquids. Fluids of non-Newtonian are not subject to the viscosity relation of Newton. Fluids of non-Newtonian have non-linear relevance between shear stress and rate of shear. Non-Newtonian fluids are further categorised into two major categories; fluids of shear thickening and fluids of shear thinning. Fluids of non-Newtonian are commonly used in mechanical and chemical industries, and also biological sciences. It has sparked the curiosity of numerous investigators who are curious in the flow of blood, flow of lubricant, and plasma flux. Many fluid paradigms have been developed to display the real fluids nature based on viscosity. These fluid models are useful for gaining a better knowledge of the rheological characteristics of non-Newtonian fluids. Among these fluids are fluids of Carreau, Maxwell, Williamson, Casson, Jaffrey, etc. There was no good mathematical model that obeys the flow of shear thinning fluids (pseudoplastic). Williamson^1^ was a pioneer in studying materials of pseudoplastic and suggesting a fluid regime for fluids of non-Newtonian, which was eventually named after him. This paradigm was submitted in 1929. Williamson fluid owing to moving surface by taking viscous dissipation into account was investigated by Megahed^2^. He elaborated that velocity of the surface was lowered by slip velocity, magnetic domain, phenomena of suction, and thickness of momentum boundary layer by solving advanced equations. The flow of MHD Williamson fluid on an extending sheet with the thermal conditions and velocity impacts was scrutinized by Lund et al.^3^. Iqbal et al.^4^ used the Williamson model to analyse numerically the flow caused by stretching plate. Gireesha et al.^5^ investigated Williamson liquid stream in a micro-channel using wall shear characteristics. Williamson paradigm flow saturated in granules was examined by Bibi et al.^6^. The flow impact on MHD Williamson paradigm with chemical reactive and heat sink/source across a flat/curved surface was checked by Kumar et al.^7^. Because usefulness of non-Newtonian fluids, numerous investigators^8–19^ have used these models to show the true behaviour of fluids throughout the previous decade.

Surfaces which have stretching property include a significant action on the quality of final goods from a manufacturing and industrial standpoint. Stretching sheet is used in a variety of applications, including paper manufacturing, wires drawing, hot rolling, plastic film drawing, metals spinning, fibre production, and extrusion. Crane^20^ was the first to introduce the notion of flux along a stretchable sheet. He talked about relocating the heat impacts caused by the flux generated by the stretched sheet. 3D MHD Casson liquid flux via permeable linearly extending slab was checked by Nadeem et al.^21^. The influences of thermal ray, changeable magnetic field and permeability which non-uniform on flux-motivated by extending sheet were discussed by Nayak et al.^22^. The pioneering study of Williamson flux across a stretchable plate by incorporating the impacts of thermal radiation, viscous dissipative and changing characteristics was done by Megahed^23^. Waini et al.^24^ investigated stream of hybrid nano-fluid mixed convection through an extendable sheet. The MHD flux of Williamson nanoliquid via an exponentially extending slab was checked numerically by Ahmed and Akbar^25^. On account of its extensive usage applications in industry, many researchers^25–28^ are drawn to explore the flow caused by stretching plate.

Discussions on the issue of boundary layer with convection conduction factor have recently sparked the curiosity of investigators due to their significance in industrial and technological fields for adjusting thermal impacts in manufacturing outputs such as systems of engine cooling computer energy supplies and devices of electronic. Aziz^29^ was the first to introduce the notion of convective boundary case. This problem has been expanded by Bataller^30^ to include radiation of thermal by taking into regard Blasius and Sakiadis flow. The factor of convective boundary on nano-fluids flux through a sheet was examined by Khan and Gorla^31^. The flux of free convection from a perpendicular non-Darcy porous material with parameter of convective boundary was elaborated by Murthy et al.^32^. Through a vertical sheet, RamReddy et al.^33^ examined the flux of mixed convection with impact of Soret and condition of convective boundary. The influence of radiation and number of Biot on the flux of nano-fluid across a flat surface was investigated by Kameswaran et al.^34^. Vasu et al.^35^ investigated entropy production and density of nonlinear temperature in stream of Newtonian liquid with a convective plate across a permeable plate.

The novelty of this article is to investigate the hybrid nanoparticles with Williamson fluid from (Ag-TiO_2_/water) formation with the effects of heat sink/source, magnetic field, thermal radiation and Ohmic heating which is not studied yet in the literature. The transformations of similarity are utilized to create the single-phase (Ag-TiO_2_/water) model and converted it to ordinary differential equations. The RKF45 method via shooting application is employed in order to produce the findings, which are validated using the numerical values from prior studies.

## Problem formulation

The 2D flux of incompressible and steady Williamson hybrid nanofluid through a stretchable curved linearly plate is studied. The surface is assumed to be wrapped in the shape of a circle with radius ${R}^{*}$ in the directions of $r-$ and $s-$, respectively. The velocity is defined as ${U}_{w}(s) = as$ with extendable constant $a > 0$. The process of heat relocation included thermal radiations and convection. The expression of stress-tensor^36^ for Williamson fluid is

1 $$\tau =\left(\frac{{\mu }_{0}-{\mu }_{\infty }}{1-\Gamma \dot{\gamma }}\right),$$

where

2 $$\dot{\gamma }=\sqrt{\frac{1}{2}\Pi },$$

3 $${\Pi } = {\text{trace}}\left( {{\text{A}}_{1}^{2} } \right),$$

after employing the approximation of boundary layer, the corresponding formulas for mass conservation, momentum, and energy are^37^:

4 $$\frac{\partial \left[\left(r+{R}^{*}\right){u}_{r}\right]}{\partial r}+{R}^{*}\frac{\partial \left({u}_{s}\right)}{\partial s}=0,$$

5 $$\frac{{u}_{s}^{2}}{r+{R}^{*}}=\frac{1}{\rho }\frac{\partial P}{\partial r},$$

6 $${u}_{r}\frac{\partial {u}_{s}}{\partial s}+\frac{{R}^{*}}{r+{R}^{*}}{u}_{s}\frac{\partial {u}_{s}}{\partial s}+\frac{{u}_{s}{u}_{r}}{r+{R}^{*}}=-\frac{1}{{\rho }_{hnf}}\frac{{R}^{*}}{r+{R}^{*}}\frac{\partial p}{\partial s}+{\nu }_{hnf}^{*}\frac{{\partial }^{2}{u}_{s}}{\partial {r}^{2}}+\Gamma \frac{{\nu }_{hnf}^{*}}{r+{R}^{*}}{\left(\frac{\partial {u}_{s}}{\partial r}-\frac{{u}_{s}}{r+{R}^{*}}\right)}^{2}+2{\nu }_{hnf}^{*}\Gamma \left(\frac{\partial {u}_{s}}{\partial r}\frac{{\partial }^{2}{u}_{s}}{\partial {r}^{2}}-\frac{{u}_{s}}{r+{R}^{*}}\frac{{\partial }^{2}{u}_{s}}{\partial {r}^{2}}+\frac{2{u}_{s}}{{\left(r+{R}^{*}\right)}^{2}}\frac{\partial {u}_{s}}{\partial s}-\frac{1}{r+{R}^{*}}{\left(\frac{\partial {u}_{s}}{\partial s}\right)}^{2}-\frac{{u}_{s}^{2}}{{\left(r+{R}^{*}\right)}^{3}}\right)-\frac{{\sigma }_{hnf}{B}_{0}^{2}}{{\rho }_{hnf}}{u}_{s}-\frac{{\nu }_{hnf}}{{K}_{1}}{u}_{s},$$

7 $${u}_{r}\frac{\partial T}{\partial r}+\frac{1}{r+{R}^{*}}{u}_{s}\frac{\partial T}{\partial s}=\frac{{k}_{hnf}}{(\rho {c}_{p}{)}_{hnf}}\left(1+\frac{16{\sigma }^{*}{T}_{\infty }^{3}}{3{k}^{*}k}\right)\left(\frac{1}{r+{R}^{*}}\right)\left[\frac{\partial T}{\partial r}+\left(r+{R}^{*}\right)\frac{{\partial }^{2}T}{\partial {r}^{2}}\right]+\frac{{Q}_{0}}{(\rho {c}_{p}{)}_{hnf}}\left(T-{T}_{\infty }\right)+\frac{{\sigma }_{hnf}{B}_{0}^{2}}{{\left(\rho {c}_{p}\right)}_{hnf}}{u}_{s}^{2},$$

the boundary conditions are:

8 $$\left.\begin{array}{c}{u}_{s}={U}_{w}=as, {u}_{r}=0, {k}_{hnf}\frac{\partial T}{\partial r}=-{h}_{f}\left({T}_{w}-T\right) at r=0,\\ \frac{\partial {u}_{s}}{\partial r}\to 0, {u}_{s}\to 0, T\to {T}_{\infty } as r\to \infty .\end{array}\right\},$$

where $${\nu }_{hnf}^{*}=\frac{{\mu }_{0}-{\mu }_{\infty }}{{\rho }_{hnf}}$$.

The following similarity transformations are:

9 $$\begin{gathered} u_{r} = - \frac{{R^{*} }}{{r + R^{*} }}\sqrt {a\nu^{*} } f\left( \eta \right),\quad u_{s} = asf^{\prime}\left( \eta \right),\quad p = \rho a^{2} s^{2} P\left( \eta \right),\quad \theta \left( \eta \right) = \frac{{T - T_{\infty } }}{{T_{w} - T_{\infty } }}, \hfill \\ \eta = \sqrt {\frac{a}{{\nu^{*} }}} r. \hfill \\ \end{gathered}$$

By using Eq. (9) in Eqs. (4)–(8), we obtain

10 $${P}^{^{\prime}}=\frac{1}{\eta +{\alpha }_{1}}{f{^{\prime}}}^{2},$$

11 $$2{\alpha }_{1}\frac{\rho }{{\rho }_{hnf}}P={\alpha }_{1}f{f}^{{^{\prime}}{^{\prime}}}-{\alpha }_{1}{\left({f}^{{\prime}}\right)}^{2}+\frac{{\alpha }_{1}}{\left({\alpha }_{1}+\eta \right)}f{f}^{{\prime}}+\frac{{\nu }_{hnf}^{*}}{{\nu }^{*}}\left({\alpha }_{1}+\eta \right){f}^{{{\prime}}{{\prime}}{{\prime}}}+\frac{{\nu }_{hnf}^{*}}{{\nu }^{*}}{\mathrm{\alpha }}_{2}{Re}^{1/2}\left[2\left({\alpha }_{1}+\eta \right){f}^{{{\prime}}{{\prime}}}{f}^{{{\prime}}{{\prime}}{{\prime}}}-2{f}^{{\prime}}{f}^{{{\prime}}{{\prime}}{{\prime}}}+\frac{2}{\left({\alpha }_{1}+\eta \right)}{f}^{{\prime}}{f}^{{{\prime}}{{\prime}}}-{\left({f}^{{{\prime}}{{\prime}}}\right)}^{2}-\frac{{\left({f}^{{\prime}}\right)}^{2}}{{\left({\alpha }_{1}+\eta \right)}^{2}}\right]-\frac{{\sigma }_{hnf}/{\sigma }_{f}}{{\rho }_{hnf}/{\rho }_{f}}M{f}^{{\prime}}-\frac{{\nu }_{hnf}}{{\nu }_{f}}K{f}^{{\prime}},$$

12 $$\left(1+\frac{4}{3}R\right)\left[\frac{\theta^{{\prime}}}{{\alpha }_{1}+\eta }+\theta^{{{\prime}}{{\prime}}}\right]+\frac{{\alpha }_{1}}{{\alpha }_{1}+\eta }Prf{\theta }^{{\prime}}+\frac{(\rho {c}_{p}{)}_{f}}{(\rho {c}_{p}{)}_{hnf}}PrS\theta +MEcPr\frac{{\sigma }_{hnf}/{\sigma }_{f}}{{\left(\rho {c}_{p}\right)}_{hnf}/{\left(\rho {c}_{p}\right)}_{f}}{{f}^{{\prime}}}^{2}=0,$$

replacing pressure $P$ from Eqs. (10) and (11), we get

13 $$\left[-\frac{{\nu }_{hnf}^{*}}{{\nu }_{f}^{*}}\left({\alpha }_{1}+\eta \right)+{2\mathrm{\alpha }}_{2}{Re}^{1/2}{f}^{{\prime}}-{2\mathrm{\alpha }}_{2}{Re}^{1/2}\left({\alpha }_{1}+\eta \right){f}^{{{\prime}}{{\prime}}}\right]{f}^{iv}={\alpha}_{1}f{{f}^{{{\prime}}{{\prime}}{{\prime}}}}-{\alpha }_{1}{f}^{{\prime}}{f}^{{{\prime}}{{\prime}}}-{\alpha }_{1}{\left({\alpha }_{1}+\eta \right)}^{-2}f{f}^{{\prime}}+{\alpha }_{1}{\left({\alpha }_{1}+\eta \right)}^{-1}{{f}^{{\prime}}}^{2}-2{\alpha }_{1}\frac{\rho }{{\rho }_{hnf}}\frac{1}{\eta +{\alpha }_{1}}{{f}^{{\prime}}}^{2}+{\alpha }_{1}{\left({\alpha }_{1}+\eta \right)}^{-1}f{f}^{{{\prime}}{{\prime}}}+\frac{{\nu }_{hnf}^{*}}{{\nu }^{*}}{f}^{{{\prime}}{{\prime}}{{\prime}}}+\frac{{\nu }_{hnf}^{*}}{{\nu }^{*}}{\mathrm{\alpha }}_{2}{Re}^{1/2}\left[2\left({\alpha }_{1}+\eta \right){{f}^{{{\prime}}{{\prime}}{{\prime}}}}^{2}-4{\left({\alpha }_{1}+\eta \right)}^{-2}{f}^{{\prime}}{f}^{{{\prime}}{{\prime}}}+2{\left({\alpha }_{1}+\eta \right)}^{-1}{{f}^{{{\prime}}{{\prime}}}}^{2}+2{\left({\alpha }_{1}+\eta \right)}^{-1}{f}^{{\prime}}{f}^{{{\prime}}{{\prime}}{{\prime}}}-2{f}^{{{\prime}}{{\prime}}}{f}^{{{\prime}}{{\prime}}{{\prime}}}+2{{\left({\alpha }_{1}+\eta \right)}^{-3}\left({f}^{^{\prime}}\right)}^{2}\right]-\frac{{\sigma }_{hnf}/{\sigma }_{f}}{{\rho }_{hnf}/{\rho }_{f}}M{f}^{{{\prime}}{{\prime}}}-\frac{{\nu }_{hnf}^{*}}{{\nu }_{f}^{*}}K{f}^{{{\prime}}{{\prime}}},$$

with boundary conditions:

14 $$\left.\begin{array}{c}f\left(0\right)=0, {f}^{{\prime}}\left(0\right)=1,\frac{{k}_{hnf}}{{k}_{f}}{\theta }^{{\prime}}\left(0\right)=-Bi\left[1-\theta \left(0\right)\right],\\ {f}^{{\prime}}\left(\infty \right)\to 0, {f}^{{{\prime}}{{\prime}}}\left(\infty \right)\to 0,\theta \left(\infty \right)\to 0.\end{array}\right\},$$

where ${\alpha }_{1}={R}^{*}\sqrt{\frac{a}{{\nu }^{*}}}$, ${\alpha }_{2}=a\Gamma$, $Re=\frac{a{s}^{2}}{{\nu }^{*}}$, $R= \frac{4{\sigma }^{*}{T}_{\infty }^{3}}{3{k}^{*}k}$,$M=\frac{{\sigma }_{f}}{{\rho }_{f}}\frac{{B}_{0}^{2}}{a}$, $K=\frac{{\nu }_{f}}{{aK}_{1}}$, $S=\frac{{Q}_{0}}{a(\rho {c}_{p}{)}_{f}}$ and $Ec= \frac{{a}^{2}{s}^{2}}{\left({T}_{w}-{T}_{\infty }\right){\left({c}_{p}\right)}_{f}}$ refer to the curvature parameter, Williamson parameter, Reynold number, thermal radiation, parameter of magnetic, porous media permeability, sink/source of heat and Eckert number.

Coefficient of skin friction $({c}_{f} )$ and local Nusselt number $(N{u}_{s})$ are defined as:

15 $${C}_{f}=\frac{{\tau }_{w}}{\frac{1}{2}\rho {u}_{s}^{2}}, Nu=\frac{s{q}_{w}}{\left({T}_{w}-{T}_{\infty }\right){k}_{f}},$$

where the wall shear stress $\left({\tau }_{w}\right)$, and heat flux $\left({q}_{w}\right)$ are:

16 $$\begin{gathered} \tau_{w} = \mu_{hnf} \left\{ {\left( {\frac{{\partial u_{s} }}{\partial r} - \frac{{u_{s} }}{{r + R^{*} }}} \right) + 2\Gamma \left( {\frac{{\partial u_{s} }}{\partial r} - \frac{{u_{s} }}{{r + R^{*} }}} \right)\left[ {\left( {\frac{{u_{r} }}{{r + R^{*} }} + \frac{{\partial u_{s} }}{\partial s}} \right)^{2} + \frac{1}{4}\left( {\frac{{\partial u_{s} }}{\partial r} - \frac{{u_{s} }}{{r + R^{*} }}} \right)^{2} } \right]^{1/2} } \right\}, \hfill \\ q_{w} = - k_{hnf} \left( {\frac{\partial T}{{\partial r}}} \right)_{r = 0} - \frac{{16\sigma^{*} T_{\infty }^{3} }}{{3k^{*} }}\left( {\frac{\partial T}{{\partial r}}} \right)_{r = 0} , \hfill \\ \end{gathered}$$

eventually, Eq. (15) in nondimensional form become:

17 $${{Re}^{1/2}C}_{f}=\frac{2{\mu }_{hnf}}{{\mu }_{f}}\left\{\left(f^{{{\prime}}{{\prime}}}\left(0\right)-\frac{f^{{\prime}}\left(0\right)}{{\alpha }_{1}}\right)+2{\mathrm{\alpha }}_{2}\left(f^{{{\prime}}{{\prime}}}\left(0\right)-\frac{f^{{\prime}}\left(0\right)}{{\alpha }_{1}}\right){\left[{\left(\frac{f\left(0\right)}{{\alpha }_{1}}-f^{{\prime}}\left(0\right)\right)}^{2}+\frac{1}{4}{Re\left(f^{{{\prime}}{{\prime}}}\left(0\right)-\frac{f^{{\prime}}\left(0\right)}{{\alpha }_{1}}\right)}^{2}\right]}^{1/2}\right\},$$

18 $${Re}^{-1/2}Nu=-\left[\frac{{k}_{hnf}}{{k}_{f}}+\frac{4}{3}R\right]{\theta }^{{\prime}}\left(0\right),$$

## Computational procedure

The controlling regime of Eqs. (12) and (13) is connected and highly non-linear. The Runge Kutta Fehlberg (RKF45) cum technique of shooting is applied to solve the system numerically for a variety of parameter values. The action of involved diverse variables on the physical quantities as $f {^{\prime}}(\eta )$, $\theta (\eta )$, ${{Re}^{1/2}C}_{f}$, and ${Re}^{-1/2}Nu$ are displayed pictorially. The precision is up to the 5^th^ decimal point as the criterion of convergence and the step size is taken as $\Delta \eta =0.01$. Against the boundary condition of far-field, we assumed an acceptable finite value in (14), that is $\eta \to \infty$, let us say ${\eta }_{\infty }$.

19 $${f}^{{\prime}}\left({\eta }_{\infty }\right)={f}^{{{\prime}}{{\prime}}}\left({\eta }_{\infty }\right)=\theta \left({\eta }_{\infty }\right)\to 0.$$

## Results and discussion

The objective of this part is to go through the impact of diverse factors on flow conduct. Table 1 explains the formulation of hybrid nano-fluid properties that was used. The properties of thermophysical of H_2_O and the nano-particles of Ag/TiO_2_ are shown in Table 2. Table 3 depicts the relevance between the previous results and the current results. This indicates the legitimacy of the current results as well the reliability of the numerical approach used in this research. To calculate the approximate relative error $\xi$ between the current findings $({r}_{c})$ and previous results $({r}_{p})$ using the formula: $\xi =\frac{\left|{r}_{c}-{r}_{p}\right|}{{r}_{c}}\times 100\%$. Table 4 displays the diverse values of $R{e}^{1/2}{C}_{f}$ and $R{e}^{-1/2}Nu$ for diverse values of ${\varphi }_{1}$, ${\varphi }_{2}$, $K$, $Ec$, $Bi$ and ${f}_{w}$ when $M = 2.0$, $Pr=6.2$, ${\alpha }_{1}=1.7$, ${\alpha }_{2}=0.1$, $R = 0.5$, and $S=-0.1$. It has been discovered that ${\varphi }_{1}$, ${\varphi }_{2}$ and $Ec$ have the same influence on skin friction and Nusselt number. The surface friction is lowered by an improvement of porosity and Biot number, but they increase the number of Nusselt.

**Table 1:** Hybrid nanofluid thermo-physical characteristics^38^.

Characteristic	Used model
$\rho$	${\rho }_{hnf}=\left(1-{\varphi }_{2}\right)\left[\left(1-{\varphi }_{1}\right){\rho }_{f}{+\varphi }_{1}{\rho }_{{n}_{1}}\right]+{\varphi }_{2}{\rho }_{{n}_{2}}$
$\rho {c}_{p}$	${\left(\rho {c}_{p}\right)}_{hnf}=\left(1-{\varphi }_{2}\right)\left[\left(1-{\varphi }_{1}\right){\left(\rho {c}_{p}\right)}_{f}+{\varphi }_{1}{\left(\rho {c}_{p}\right)}_{{n}_{1}}\right]+{\varphi }_{2}{\left(\rho {c}_{p}\right)}_{{n}_{2}}$
$\mu$	${\mu }_{hnf}=\frac{{\mu }_{f}}{{\left(1-{\varphi }_{1}\right)}^{2.5}{\left(1-{\varphi }_{2}\right)}^{2.5}}$
$k$	${k}_{hnf}=\frac{{k}_{{n}_{2}}+2{k}_{nf}-2{\varphi }_{2}\left({k}_{nf}-{k}_{{n}_{2}}\right)}{{k}_{{n}_{2}}+2{k}_{nf}+{\varphi }_{2}\left({k}_{nf}-{k}_{{n}_{2}}\right)}\times \frac{{k}_{{n}_{1}}+2{k}_{f}-2{\varphi }_{1}\left({k}_{f}-{k}_{{n}_{1}}\right)}{{k}_{{n}_{1}}+2{k}_{f}+{\varphi }_{1}\left({k}_{f}-{k}_{{n}_{1}}\right)}\times {k}_{f}$
$\sigma$	${\sigma }_{hnf}=\frac{{\sigma }_{{n}_{2}}+2{\sigma }_{nf}-2{\varphi }_{1}\left({\sigma }_{nf}-{\sigma }_{{n}_{2}}\right)}{{\sigma }_{{n}_{2}}+2{\sigma }_{nf}+{\varphi }_{2}\left({\sigma }_{nf}-{\sigma }_{{n}_{2}}\right)}\times \left[1+\frac{3\left(\frac{{\sigma }_{n1}}{{\sigma }_{f}}-1\right){\varphi }_{1}}{2+\frac{{\sigma }_{n1}}{{\sigma }_{f}}-\left(\frac{{\sigma }_{n1}}{{\sigma }_{f}}-1\right){\varphi }_{1}}\times {\sigma }_{f}\right]$
$\beta$	${\left(\rho \beta \right)}_{hnf}=\left(1-{\varphi }_{2}\right)\left[\left(1-{\varphi }_{1}\right){\left(\rho \beta \right)}_{f}+{\varphi }_{1}{\left(\rho \beta \right)}_{n1}\right]+{\varphi }_{2}{\left(\rho \beta \right)}_{n2}$

**Table 2:** Thermophysical properties of hybrid nanofluid^39^.

Characteristic	Water	Ag	TiO_2_
${c}_{p}$($\mathrm{J}/\mathrm{kgK}$)	4179	235	686.2
$\rho$($\mathrm{kg }{\mathrm{m}}^{-3}$)	997.1	10.500	4250
$k$($\mathrm{W }{\mathrm{m}}^{-1}{\mathrm{K}}^{-1}$)	0.613	429	8.9538
$\sigma \times {10}^{-6} (\mathrm{S}/\mathrm{m})$	5.5 × 10^–12^	63	2.4

**Table 3:** Comparison of Nusselt number with Ref.^40^ while $Pr = 2.0$, and $R=0.2$.

${\alpha }_{2}$	${\alpha }_{1}$	$Re$	Ref.^40^	Present	$\xi$
			$\frac{1}{2}R{e}^{-1/2}Nu$	$\frac{1}{2}R{e}^{-1/2}Nu$	
0.1	0.6	0.1	1.5241	1.5241	0.0000
0.2			1.5121	1.5237	0.7613
0.3			1.4983	1.5235	1.6540
0.1	0. 4		1.7110	1.7258	0.8575
	0.6		1.5241	1.5241	0.0000
	1.0		1.3412	1.3505	0.6886
	0.7	0.2	1.4590	1.4634	0.3006
		0.5	1.4504	1.4630	0.8612

**Table 4:** values of $R{e}^{1/2}{C}_{f}$ and $R{e}^{-1/2}Nu$ for diverse values of ${\varphi }_{1}$, ${\varphi }_{2}$, $K$, $Ec$, $Bi$ and ${f}_{w}$ when $M = 2.0$, $Pr=6.2$, ${\alpha }_{1}=1.7$, ${\alpha }_{2}=0.1$, $R = 0.5$, and $S=-0.1$.

${\varphi }_{1}$	${\varphi }_{2}$	$K$	$Ec$	$Bi$	$-R{e}^{1/2}{C}_{f}$	$R{e}^{-1/2}Nu$
0	0.01	0.5	0.1	0.5	24.2058	0.6546
0.01					24.2090	0.6499
0.02					24.2208	0.6454
	0.03				25.7175	0.6382
	0.04				26.4754	0.6347
	0.05				27.2399	0.6312
		0.6			27.3190	0.6313
		0.7			27.3979	0.6313
		1.0			27.6328	0.6314
			0.02		27.2399	0.6464
			0.04		27.2399	0.6426
			0.08		27.2401	0.6350
				0.6	27.2399	0.7377
				0.7	27.2401	0.8339

**Figure 1:**
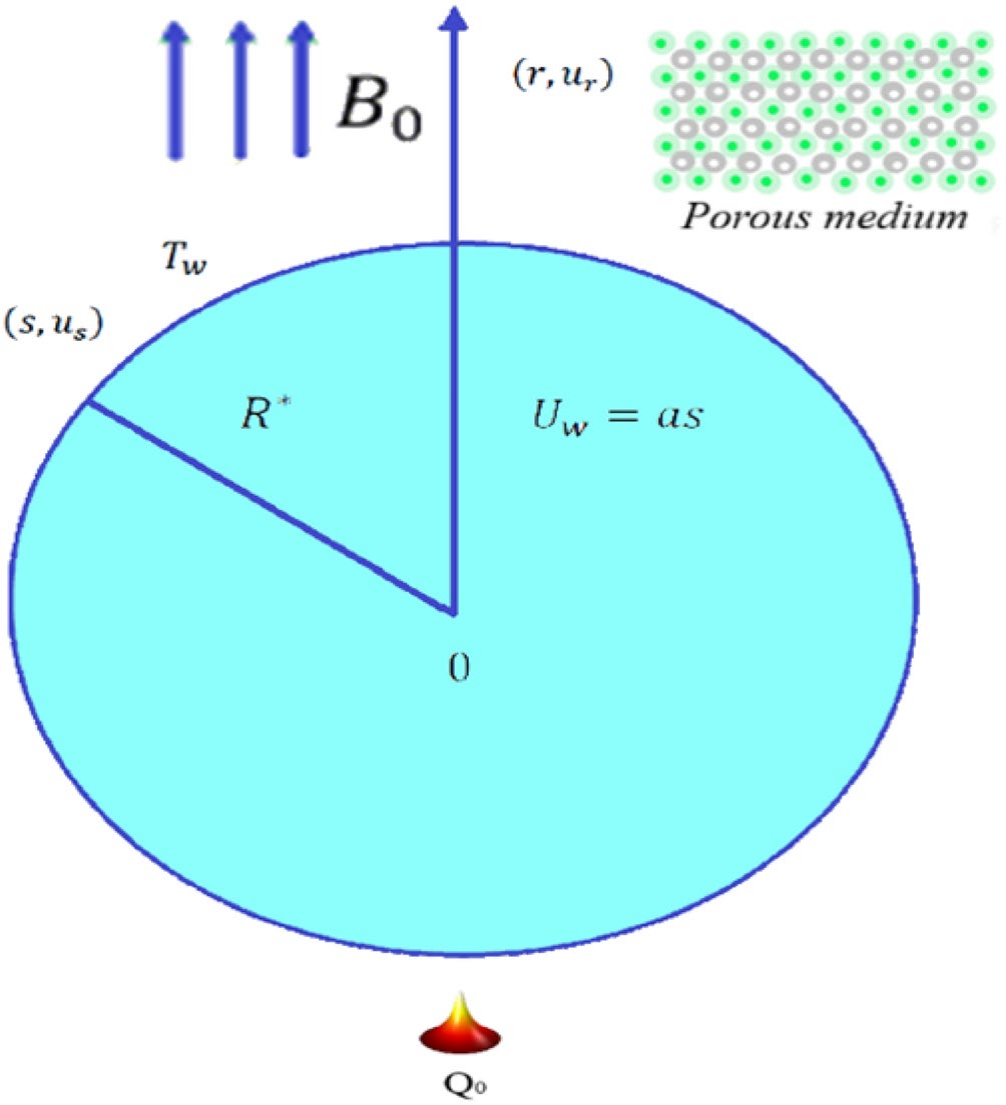
Physical representation with coordinate system.

**Figure 2:**
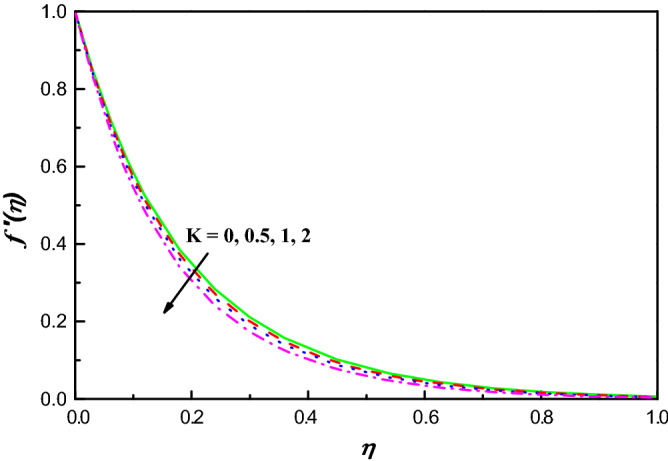
$$f {^{\prime}}\left(\eta \right)$$ variation vs. $$K$$.

**Figure 3:**
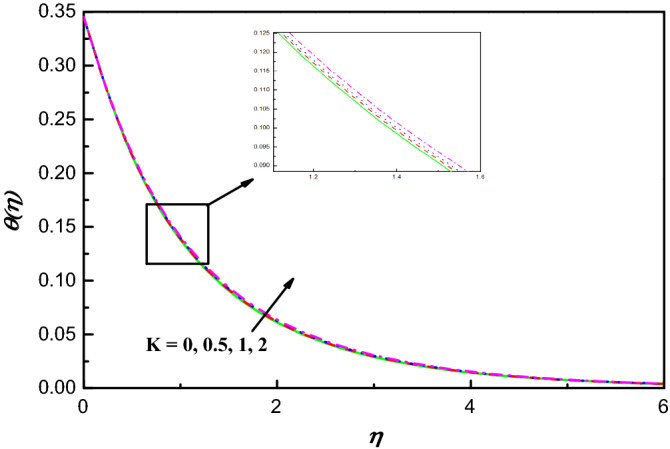
$$\theta \left(\eta \right)$$ variation vs. $$K$$.

**Figure 4:**
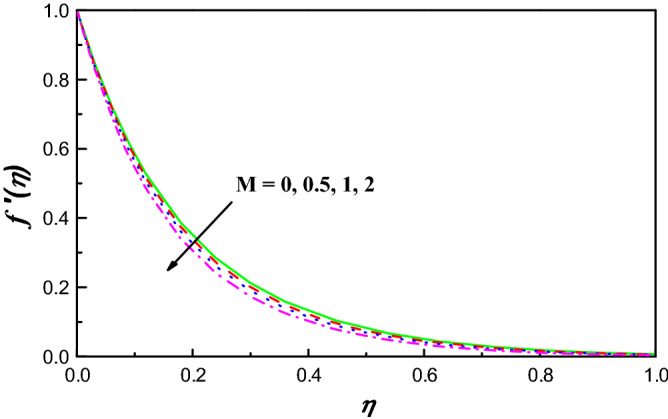
$$f {^{\prime}}\left(\eta \right)$$ variation vs. $$M$$.

**Figure 5:**
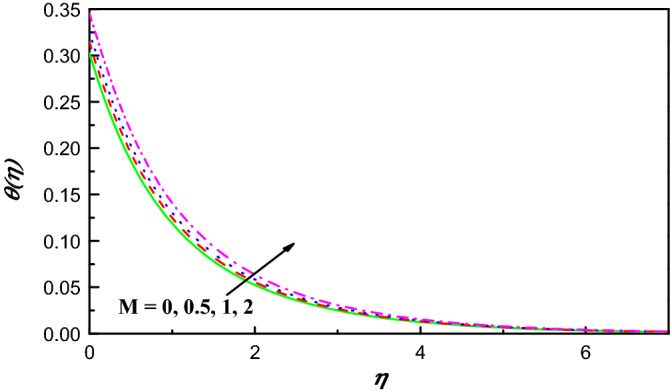
$$\theta \left(\eta \right)$$ variation vs. $$M$$.

**Figure 6:**
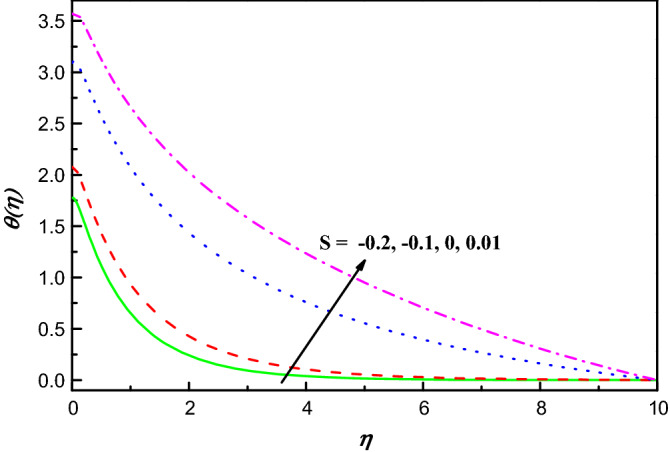
$$\theta \left(\eta \right)$$ variation vs. $$S$$.

**Figure 7:**
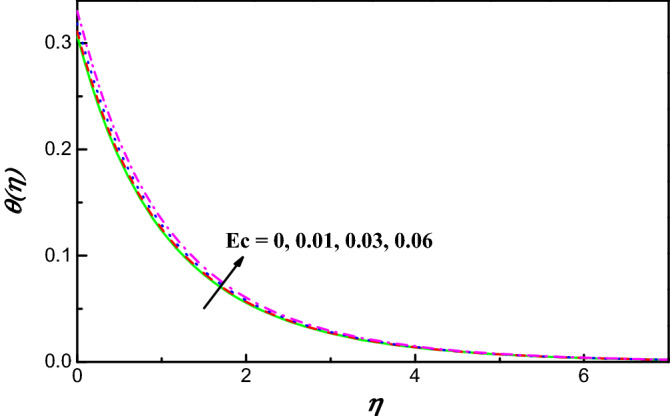
$$\theta \left(\eta \right)$$ variation vs. $$Ec$$.

**Figure 8:**
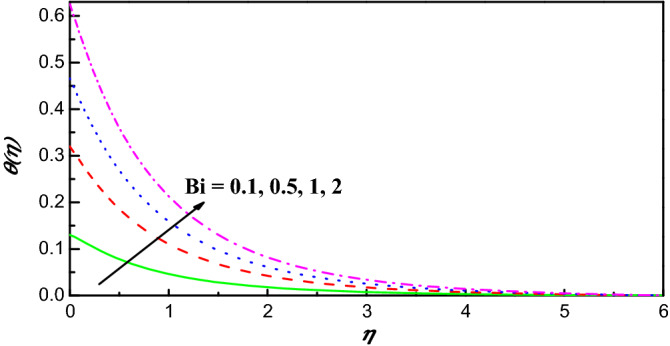
$$\theta \left(\eta \right)$$ variation vs. $$Bi$$.

**Figure 9:**
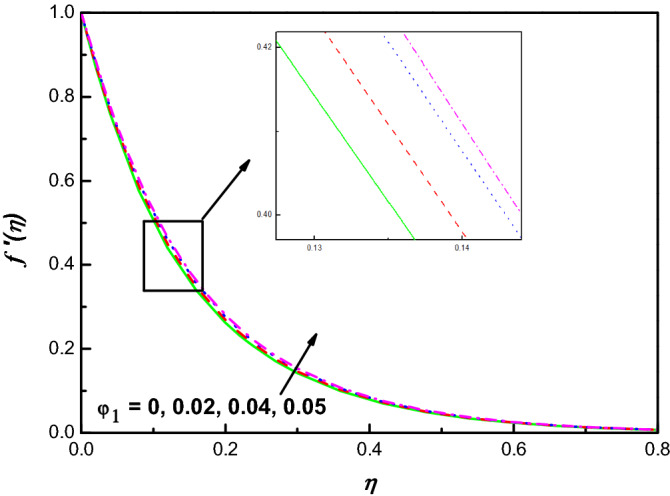
$$f {^{\prime}}\left(\eta \right)$$ variation vs. $${\varphi }_{1}$$.

**Figure 10:**
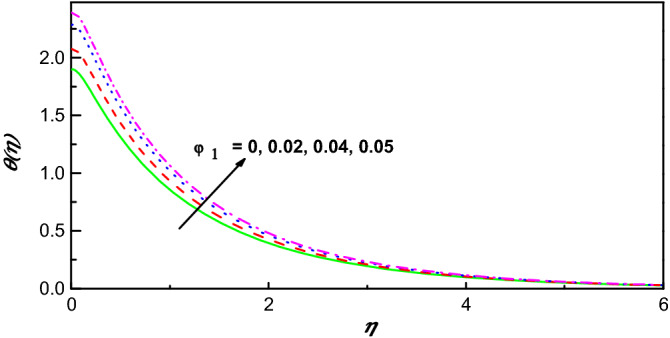
$$\theta \left(\eta \right)$$ variation vs. $${\varphi }_{1}$$.

**Figure 11:**
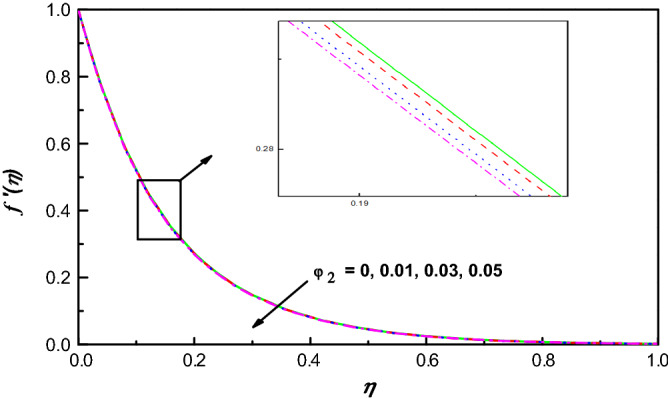
$$f {^{\prime}}\left(\eta \right)$$ variation vs. $${\varphi }_{2}$$.

**Figure 12:**
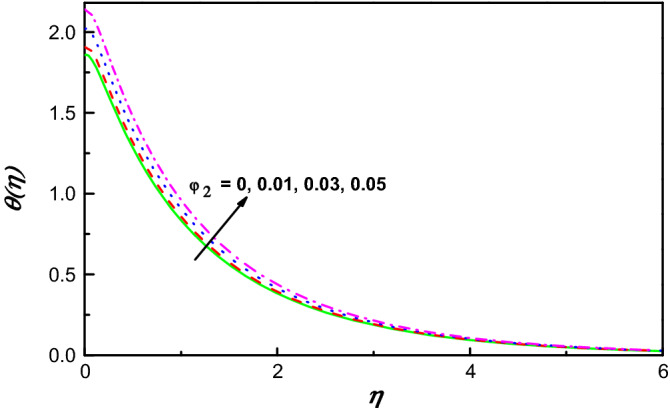
$$\theta \left(\eta \right)$$ variation vs. $${\varphi }_{2}$$.

**Figure 13:**
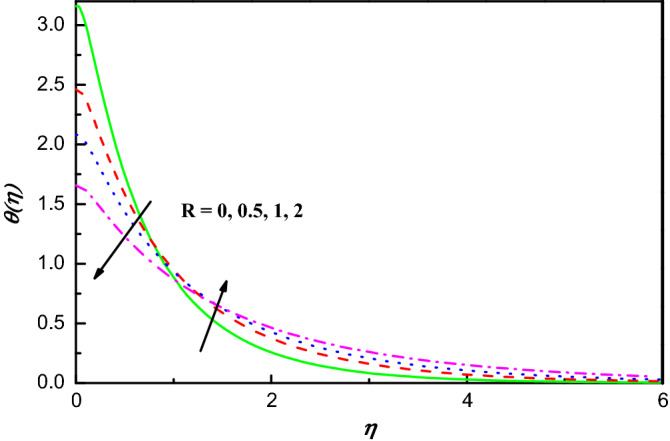
$$\theta \left(\eta \right)$$ variation vs. $$R$$.

**Figure 14:**
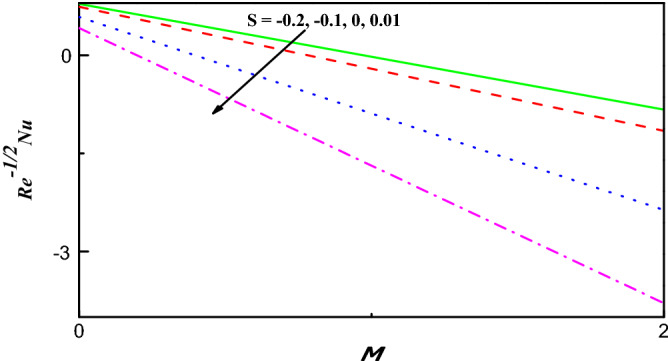
$R{e}^{-1/2}Nu$ Variation vs. $M$ with $S$.

**Figure 15:**
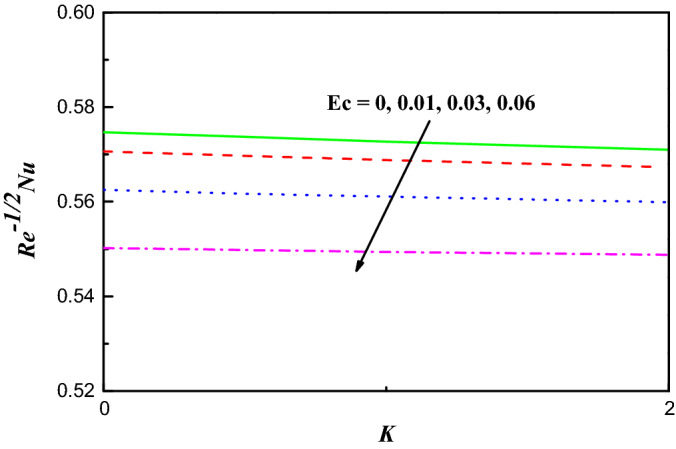
$R{e}^{-1/2}Nu$ Variation vs. $K$ with $Ec$.

**Figure 16:**
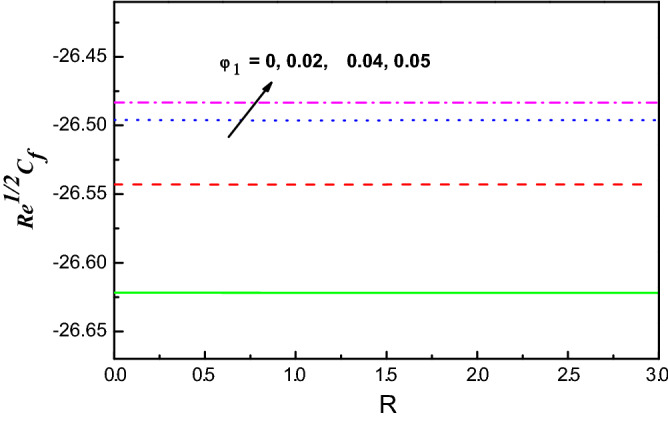
$R{e}^{1/2}{C}_{f}$ Variation vs. $R$ with ${\varphi }_{1}$.

**Figure 17:**
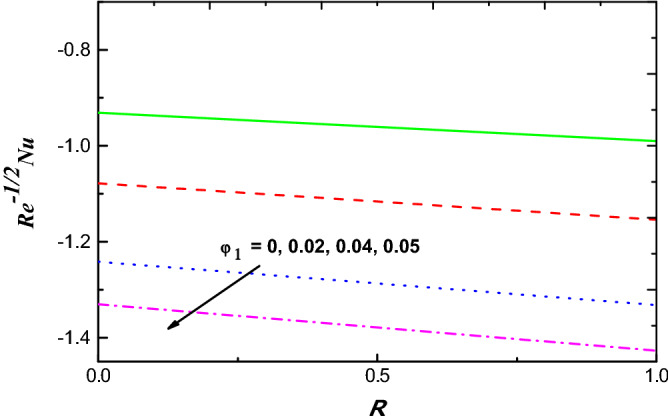
$R{e}^{-1/2}Nu$ Variation vs. $R$ with ${\varphi }_{1}$.

**Figure 18:**
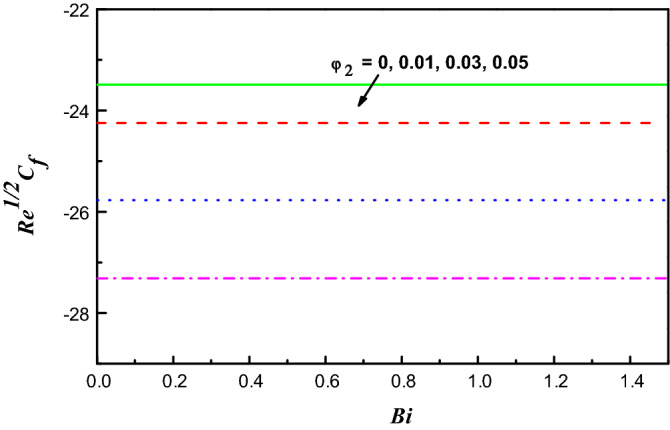
$R{e}^{1/2}{C}_{f}$ Variation vs. $Bi$ with ${\varphi }_{2}$.

**Figure 19:**
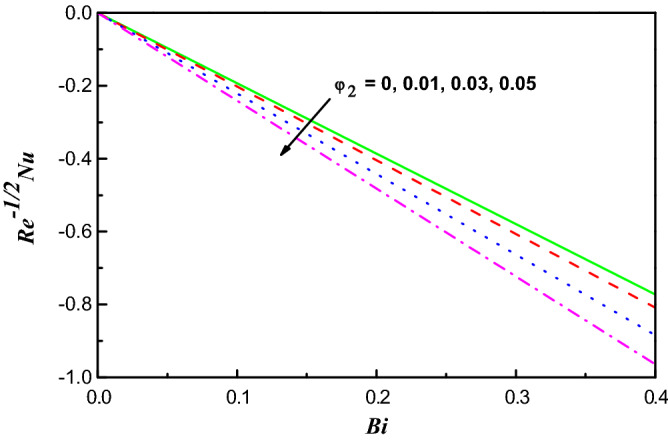
$R{e}^{-1/2}Nu$ Variation vs. $Bi$ with ${\varphi }_{2}$.

Figures 1, 2, 3, 4, 5, 6, 7, 8, 9, 10, 11, 12, 13, 14, 15, 16, 17, 18 and 19 are presented to show the effect of diverse factors on the common distributions. Figures 2 and 3 uncover the action of profiles of velocity $f{^{\prime}}\left(\eta \right)$ and temperature $\theta \left(\eta \right)$ being transferred by dimensionless $K$ parameter in Williamson hybrid nano-fluids, respectively. It is seen that $f{^{\prime}}\left(\eta \right)$ has diminishing conduct for increasing $K$ as illustrated in Fig. 2. The fact behind is that the presence of $K$ leads to upsurge the protection against the fluid's smooth motion which makes $f{^{\prime}}\left(\eta \right)$ decreases and because of which there is ascend in the distribution of temperature. This conduct of $\theta \left(\eta \right)$ is clearly observed from Fig. 3, which explains that the profile of temperature carries out an improvement with the expanding $K$ parameter.

Figure 4 exposes the effect of magnetic field on ${f}^{{\prime}}\left(\eta \right)$ over a curved extending surface. Since the field of magnetic experiences to be double, the component of the velocity caused to be lowered. The fact behind this is that when $M$ parameter is activated, it creates forces of Lorentz, which resist the fluid flow. Moreover, the momentum boundary layer thickness extremely lowers with an increment in $M$. So, the parameter of magnetic field has an important turn in the velocity profile. Figure 5 reflects the contrast of $\theta \left(\eta \right)$ profile with the field of magnetic. It is detected that increasing happens in temperature with growing values of $M$. As Lorentz force effects on $f{^{\prime}}\left(\eta \right)$ causes friction on the flux, this is the main reason for the production of great heat energy.

The temperature distribution under the action of $S$ is portrayed in Fig. 6, when other parameters are fixed. It is undeniable that when $S$ increases, $\theta \left(\eta \right)$ improves consistently. Physically, increasing $S$ parameter causes the flux to rise and makes the liquid flux within the boundary layer more sensible to heating effects at the curved plate. As a result, more heat energy is transported from the hot plate to the hybrid nanofluid, causing temperature to up-going via boundary layer as observed in Fig. 6.

Figures 7 and 8 exhibit the impact of Eckert and Biot numbers on $\theta \left(\eta \right)$, respectively. Figures 7 and 8 offer the temperature and the thickness of the associated boundary layer have against trend with heating of Ohmic and number of Biot. Owing to the great energy of kinetic which has direct relation with Eckert number the temperature upsurges as seen in Fig. 7. An enhancement in parameter of convection conduction reduces the plate heat resistance and upsurges convective transport of heat to hybrid nanoliquid as illustrated in Fig. 8. Mathematically, $\frac{{k}_{hnf}}{{k}_{f}}{\theta}{^{\prime}}=-Bi\left(1-\theta \right)$ which implies that $1+\frac{1}{Bi}\left( \frac{{k}_{hnf}}{{k}_{f}}\right){\theta}^{{\prime}}=\theta$. This implies that $\theta$ arrives to $1$ as $Bi\to \infty .$

Figures 9 and 11 illustrate the influence of nanomolecules of Ag $\left({\varphi }_{1}\right)$ and TiO_2_ $\left({\varphi }_{2}\right)$ on $f{^{\prime}}(\eta )$ respectively, when other parameters are stationary. It is illustrated that growing amounts of ${\varphi }_{1}$ increase $f{^{\prime}}(\eta )$, but increasing values of ${\varphi }_{2}$ reduces the profile of $f{^{\prime}}(\eta )$ and the thickness of the corresponding boundary-layer which may be that of greater collision between the suspended nanoparticles. The profiles of temperature under the action of Ag and TiO_2_ are portrayed in Figs. 10 and 12. From Figs. 10 and 12 for base liquid and mixture of nanoparticles, any one can observe obviously an improvement in $\theta \left(\eta \right)$ with increasing volume fraction of nanoparticle. The truth is that including nanoparticles with various volume fractions improves the thermal characteristics of the steward fluid, hence growing its temperature.

For upsurging parameter of $R$, the profile of temperature lowers initially, whereas reverse demeanor is observed when $\eta >2.6$. Physically, this is due to an excess in $R$ boosts the increment and transmission of additional heat into the flow, which aids increase the thickness of thermal boundary layer. This conduct of $\theta \left(\eta \right)$ is obviously watched from Fig. 13.

The schematics visualizations of the transport of heat conduct due to diverse amounts of $S$ and $Ec$ against $M$ and $K$ are plotted in Figs. 14 and 15, respectively. It is scrutinized that ${{Re}^{-1/2}Nu}$ is improved when $S$, $Ec$, $M$ and $K$ parameters increase as portrayed in Figs. 14 and 15. Figures 16 and 17 work with the variation of ${{Re}^{1/2}C}_{f}$ and ${Re}^{-1/2}Nu$ with respect to ${\varphi }_{1}$ parameter for diverse radiation parameter. It is noted that growing parameters of ${\varphi }_{1}$ and $R$ has different impact on ${{Re}^{1/2}C}_{f}$ and ${Re}^{-1/2}Nu$. Upsurging values of ${\varphi }_{1}$ and $R$, increases ${{Re}^{1/2}C}_{f}$ but reduces ${Re}^{-1/2}Nu$ as in Figs. 16 and 17.

Figures 18 and 19 display the distributions of ${{R{e}}^{1/2}C}_{f}$ and ${R{e}}^{-1/2}N{u}$ reverse $Bi$ for several amounts of ${\varphi }_{2}$. With growing values of ${\varphi }_{2}$ in H_2_O, it is cleared that ${R{e}^{1/2}C}_{f}$ and ${R{e}}^{-1/2}N{u}$ reduce. With upsurging $Bi$ upsurges the force of drag, but delays ${R{e}}^{-1/2}N{u}$ in this model.

## Conclusions

This study shows the implementation of thermal engineering in the vicinity of solar radiation for the investigation of Williamson hybrid nanofluid over a curved extendable plate with radius $\it {\mathrm{R}}^{*}$ under the assumption of boundary layer. The curvilinear coordinate model is introduced to model the problem. Significant aspects of change of heat phenomenon have nonlinear radiations, the effects of magnetic field, heat sink/source, Ohmic heating and convective boundary condition are presented in this study. The significant results of this study are:

- For greater values of magnetic field, permeability parameter and nanoparticles of TiO_2_, slowdown velocity.
- Any boost in parameter of heat sink/source causes an increment trend for field of temperature.
- Temperature distribution is upsurged with rising values of $M$,$K$,$Ec$, $Bi$, $R$, ${\varphi }_{1}$ and ${\varphi }_{2}$ parameters.
- Increasing coefficient of skin friction has a direct relevance with increasing ${\varphi }_{1}, R, Bi$.
- Drag force has a reverse function with solid volume fraction of TiO_2_.
- All parameters which we mentioned above have against trend with heat transfer.

## Data Availability

All data analyzed or generated during this study are included in this article.

## List of symbols

${c}_{p}$: Specific heat due to constant pressure, J/kgK
${k}_{hnf}$: Thermal conductivity of the hybrid nanofluid, W/mK
${K}_{1}$: Permeability of porous media, m^2^
$$p$$: Pressure, Pa
$$a$$: Constant
${q}_{w}$: Heat flux from the surface, W/m^2^
$$M$$: Magnetic parameter
$T$: Temperature of the hybrid nanofluid, K
${T}_{W}$: Temperature at the wall, K
$$R$$: Thermal radiation
$S$: Heat source $(S > 0)$ or sink $(S < 0)$
$$Ec$$: Eckert number
$$Nu$$: Nusselt number
$$Pr$$: Prandtl number
$$Re$$: Reynolds number

## Greek symbols

$\rho$: Density of the nanofluid, kg/m^3^
$$\sigma$$: Electrical conductivity, W/m
$\mu$: Dynamic viscosity, kg/ms
${\mu }_{0}$: Zero shear viscosity, kg/ms
${\mu }_{\infty }$: Infinite shear viscosity, kg/ms
${(\rho {c}_{p})}_{f}$: Heat capacity of the base fluid, J/km^3^
${(\rho {c}_{p})}_{p}$: Effective heat capacity of the nanoparticles, J/km^3^
$$\upsilon$$: Kinematic viscosity, m^2^/s
$${\tau }_{w}$$: Skin frictions
$\eta$: Dimensionless similarity variable
$$\theta$$: Dimensionless temperature
